# Ageing and exercise: building body capital in old age

**DOI:** 10.1186/s11556-018-0195-9

**Published:** 2018-04-28

**Authors:** Astrid Bergland, Marit Fougner, Anne Lund, Jonas Debesay

**Affiliations:** 1 Oslo and Akershus University College of Applied Sciences, Faculty of Health Sciences, Pilestredet, P.O. Box 4 St. Olavs plass, 0130 Oslo, Norway; 2 Faculty of Health Sciences, Pilestredet, P.O. Box 4 St. Olavs plass, N-0130 Oslo, Norway; 3 Faculty of health Sciences, OsloMet – Oslo Metropolitan University, P.O. Box 4 St. Olavs plass, N-0130 Oslo, Norway

## Abstract

**Background:**

Research that provides better understanding of the motivational processes in older age to maintain a healthy and active lifestyle is sought after. We apply theoretical approaches to cultural capital, active and healthy aging health to shed light on the women’s experiences in maintaining physical capabilities through an active lifestyle, and thereby facilitating their own inclusion in society. Thus, the aim of this paper is to explore why older home dwelling women over the age of 70 years or more spend time in physical exercise and their experiences about the importance of participating in group exercise for their daily life.

This paper reports on a qualitative study based on interviews with 16 older women aged 70 years or more and regularly attending group exercise classes in the community at an established workout center. The data were analyzed the data using an inductive content analysis approach.

**Results:**

Three overreaching and interrelated themes emerged from the interviews: “Building body capital for independence”, “Building body capital to maintain vitality and being in control” and “Building resources for social interaction”. The findings suggest that group exercise is important for building body capital. The group exercise helped the women in building bodily ability to manage everyday life, maintain vitality, being in control, pursue social interaction and live independently. These body resources were important for these older women’s experience of the manageability and meaningfulness of daily life.

**Conclusion:**

This study has provided insights into older women’s understanding and experiences of the challenges of everyday life within a theoretical framework of cultural capital and health. The women acquired cultural health capital, and more specifically body capital, by participating in the group exercise classes. The women’s investment in body capital through regular physical activity created resources which facilitated social participation. Therefore professionals need to be aware of this when performing group exercise.

## Background

A growing body of literature suggests that physical exercise has beneficial effects on physical, psychological and social functions in healthy older adults [1–3]. Research generally supports the mood-boosting, anxiety-reducing, stress-buffering and self-esteem-generating benefits of physical activity [4, 5]. Evidence about health promotion targeting particularly exercise in older age is also mounting [6–9]. The outcomes of active aging contribute positively both to individuals’ health and social expenditure [10]. The beneficial aspects of healthy aging or active aging are therefore gaining currency, as is clearly put forward in the World Report on Ageing and Health, launched in 2015 by the World Health Organization [3], where it is suggested that there has been a shift in focus from diseases and morbidity to functional ability [2, 11]. Research shows that a life of physical inactivity leads to increased morbidity and mortality, and that physical activity promotes and preserves health [3, 12]. Although increasing one’s physical capabilities has predominantly positive connotations, participation in physical activity by people over a certain age challenges popular notions of aging as an inevitable decline or as a negative process [13–17].

The aim of this current study is to explore why older home dwelling women over the age of 70 years or more spend time on group exercise and their experiences of the importance of group exercise for their daily life. We apply theoretical approaches to cultural capital, active and healthy aging to shed light on the women’s experiences in maintaining physical capabilities through an active lifestyle, and thereby facilitating their own inclusion in society.

### Theoretical framework

The WHO defined active and healthy aging as the process of optimizing opportunities for health, participation, and security in order to enhance the quality of life as people age.

Usually, the term “healthy” refers to physical, mental, and social well-being [18]. “Successful aging,” commonly used in gerontology and geriatrics, refers to the optimization of life expectancy while minimizing physical and mental deterioration and disability. It focuses on the absence or avoidance of disease and risk factors for disease, maintenance of physical and cognitive functioning, and active engagement with life (including maintenance of autonomy and social support). Some investigators have broadened the model to include more psychosocial elements, such as life satisfaction, social participation, and functioning, and psychological resources, including personal growth, resolution and fortitude, happiness, relationships between desired and achieved goals, self-concept, mood, and overall wellbeing.

Successful aging is seen as a dynamic process, as the outcome of one’s development over the life course [18]. Inspired by Pierre Bourdieu, ‘cultural capital’ is increasingly being used as a conceptual tool in the understanding of health behavior. Bourdieu’s notion of cultural capital encompasses diverse cultural practices as forms of cultural capital or resources. These resources are learned experiences from the past, and as such they are dispositions that direct and shape present behavior [19, 20]. These resources are learned experiences from the past, and as such, they are dispositions that direct and shape present behavior [19, 20].

Abel [21] points out that cultural factors become important in health promotion, including exercise programs for physical activity, when people themselves are increasingly expected to maintain their health in their everyday lives. Cultural factors heavily influence health promotion behavior because health is not a natural given, but must be acquired and maintained throughout the life course [20]. Health relevant behavior is furthermore associated with broader societal values such as body perceptions, and they are socially learned.

Consequently, this behavior has been turned into cultural assets, e.g. health promoting lifestyle, being incorporated inside the body and serve as cultural capital in everyday life. These health behaviors can be conceptualized as invested and accumulated cultural resources. The acquisition and maintenance of these resources depend on the enjoyment of the activity and require a significant personal time investment. Choosing a lifestyle with regular physical activity is therefore a form of cultural capital. This cultural capital can be transformed or applied to achieve physical health gains, but also other socially desirable means derived from those health gains, such as social belonging [21].

While some of the theoretical approaches using the term “cultural capital” focus more generally on health, others underscore the specific significance of the body as capital. In this regard, Pinxten and Lievens [22] and Antoninetti and Garrett [23], referring to Bourdieu’s three forms of capital (economic, cultural and social), draw attention on the need to expand this to include the “full self” and the “body capital” as such a component. Furthermore, Antoninetti and Garret [23] suggest the following definitorial remark for the body capital component: the individual’s capabilities to manoeuvre in the field of bodily behavior dictated by “personal characteristics of body functions and socially constructed external opinions and attitudes toward” those abilities or disabilities. Notably, diminishing bodily capabilities or the experiences of aging can be mitigated through investment in body capital. Therefore, as Eman [24] suggests, the body might be conceptualized as a site of capital accumulation or an investment opportunity. Additionally, accumulated cultural health capital serves as a tool kit of resources that can be used to create strategies for action. Such action may include health related practices like physical activity [19].

## Methods

### Study design

This qualitative study is informed by the interpretative tradition of hermeneutics and phenomenology. The exploration and utilization of the various preconceptions arising between the participant and researcher play an important part in facilitating understanding [25, 26].

### The participants

The participants taking part in the study are women over the age of 70 years or more who regularly attend group exercise classes at an established workout center for women that represents an alternative to the large fitness chains. The majority of the users are adults between 45 and 65 years, but the center also has various exercise programs for women aged 70 years and older, of whom the oldest participant is 93 years old. The center offers classes of 60 min duration for older adults and senior women twice a week. There are usually about 15 participants in each of the two classes, and some women attend both.

At the request of one of the authors with knowledge of the senior class, the exercise center staff recruited participants by informing the senior classes about the study. Eighteen participants registered their interest in participating in the study, but two of them declined to partake when contacted. Sixteen informants ranging from 70 to 85 years old, the average age being 74.8 years, were interviewed individually (Table 1).

**Table 1:** The characteristics of the informants

Informant	Age	Level of education	Living alone	Disease/type of problems
1	72	12 years or less	No	None
2	85	More than 12 years	Yes	Osteoarthritis knees, hands and shoulder
3	76	More than 12 years	Yes	Osteoporosis
4	70	More than 12 years	No	Increased level of cholesterol
5	70	12 years or less	No	High blood pressure
6	74	More than 12 years	Yes	Muscular rheumatism, hip fracture, broken arm
7	81	12 years or less	Yes	High blood pressure
8	75	12 years or less	No	None
9	72	More than 12 years	Yes	Knees osteoarthritis/high blood-pressure.
10	81	More than 12 years	Yes	High blood pressure
11	70	12 years or less	No	None
12	71	12 years or less		Ostheoarthritis
13	72	More than 12 years	Yes	Glaucoma
14	78	More than 12 years	Yes	Knees osteoarthritis chronic lymphocytic leukemia/Former breast cancer
15	73	More than 12 years	Yes	Knees osteoarthritis/Shoulder prosthesis
16	77	More than 12 years	Yes	Ulcerative colitis hypothyroidism incontinence

### The content of the group exercise classes

A female instructor, who explains, demonstrates and performs exercises leads the class supporting the participants individually and collectively. The aim is to provide functional training, to increase body awareness and to promote the enjoyment of agility exercise. Agility is the ability to change the direction of the body in an efficient and effective manner requiring a combination of balance, speed, strength and coordination [27]. The key to supporting and inspiring performance and to enjoying movement in the exercise class is the use of music, including jazz, classical music, ethnic and soul, pop and tango. Segments of music are tailored to various components of the exercise session matching with tempo movements and the intensity of work out. The activities are performed at a moderately intense level for 60 min. The program consist of weight bearing resistance, balance and stretching exercises, incorporating dancing steps and movements. Exercise equipment includes barre, providing support during various types of exercises and external loads including light hand weights, resistance band or cuffs. The program is varied and with few and short breaks. The participants are informed by their instructors about recommended guidelines for moderate intensity: “your breathing quickens, but you’re not out of breath. You develop a light sweat after about 10 minutes of activity. You can carry on a conversation, but you can’t sing”. Based on these cues the participants are encouraged to adjust the intensity to a preferred intensity without going outof your comfort zone. The exercise programs were performed at moderate level of intensity.

### Interviews

The face to face interviews were conducted in the office of two of the authors (AB & MF).

The authors interviewed the participants from half the group each.We developed an interview guide for this study, see Table 2, which included questions concerning the participants’ experiences of the physical exercise while attending the program. We asked about the influence the exercise had had on their everyday life regarding physical, psychological and social capabilities and whether they had positive or negative experiences with the exercise and how they perceived themselves and others engaging in the exercise. The interview guide was based on existing literature and our own experiences as professionals leading group exercise.

**Table 2:** Interview guide

Can you please describe your experiences with the group exercise?	
Can you please describe your negative experiences as well as your positive experiences?	
Can you please describe what group exercise means to your everyday life?	
Can you please describe a typical 1-h group exercise?	
Can you please describe how do you experience yourself and others during the group exercises?	
Can you please describe your motivation for participation in the group exercises?	
Is there anything else you want to tell me, something you find important?	
*Follow-up questions:* Could you please explain…? Could you tell me more about…?	

### Analysis

All of the interviews were voice recorded and each lasted approximately one hour. The interviews were transcribed by the interviewers. The data were analyzed using an inductive content analysis and performed collaboratively by all the authors. The inductive approach enables researchers to identify key themes in the area 182 of interest by reducing the material to a set of themes or categories [28]. The intention was to provide a compact yet general description of the phenomenon under investigation. The themes emerged from the raw data by repeated examination and comparison. Using an analytic process, we experienced an increased understanding of the material describing the participants’ experiences with the group exercise program. To ensure the trustworthiness of our analyses, as all qualitative group exercise program. To ensure the trustworthiness of our analyses, as all qualitative analyses entail a degree of interpretation, the four authors read the transcripts independently several times. Following Brinkmann and Kvale [29], the reading of transcripts was performed with an open mind so as to grasp the participants’ own views on the subject. Additional analysis was performed using the following procedures: 1) The transcripts were read to gain a contextualized impression of the text and previous preconceptions were highlighted. 2) Units of meaning were identified and coded and agreement between all four authors was based on consensus after discussion. 3) The meaning in the coded groups was then condensed. 4) The descriptions reflecting the participants’ experiences were generalized into categories and consensus regarding the categorization of statements and emerging themes were reached by discussion between all four authors. The analyses process is described in Table 3. The inductive content analyses eventually led to the use of the theoretical lens of cultural capital, which enhanced our understanding of the women’s experiences participating in the group exercise.

Mapping preconception is a key aspect of qualitative research [30, 31] and highlights the aspects of the researchers’ preconceptions relevant to the study. At the time of the interviews and analysis, our immediate preconceptions were very much linked to the experiences of all authors working and doing research among older persons living in at home and health promotion. Instead of bracketing these, they enabled us to challenge some of the interviewees’ statements and descriptions. These preconceptions were challenged and discussed throughout.

**Table 3:** Analysis process regarding three overarching themes

Themes	*Building body capital for independence*	*Building body capital to maintain vitality and be in control*	*Building body capital for social interaction*
Codes/subthemes	Everyday challenges Health management Views on future Lifelong habits Compensating ageing related changes	Feeling power and strength Improved self-esteem Health promoting habits	Increased social activity Positive feedback Sense of safety Stamina
Illustrative quotes	The exercise gives me an experience of connection between body and mind. It helps to reduce unease and sadness. It improves self-esteem and attention. I think the training contributes to good health and maintenance of it. I find that I am able to do things that are important to me.	The body carries me through everyday life and allows me to have many nice experiences. Obviously, the feelings of resignation may take over sometimes, but there is much to gain from being conscious on challenging the body, being aware of posture and facial expressions. I think I’d say it’s sometimes about saying to yourself “up with your chin.” It’s an old experience. A straight body posture gives you a more energetic experience.	I get a lot of praise for it, not least from those who do not know that I am exercising, but who guess that I’m engaged in something. It’s nice, I actually like very much being visible [laughs]. By feeling physically strong through the exercise, the women also gained a sense of safety and could easily engage in social activities.

### Ethics

This study has been approved by the Data Protection Official for Research at The Norwegian Social Science Data Services, reference number 41165. All the participants received written information on the purpose and methods of the study, provided their consent to participation in the study and were informed of the right to refuse to participate at any stage in the study.

## Results

Most of the participants in our study (see Table 2) had some health problems. However, they were living independently and were coping with everyday life without assistance from health services. Approximately 2/3 of them were living alone and 60% had more than 12 years of education. They had participated in exercise for more than twenty years and thought exercise was of importance for maintaining bodily strength as well as building resources for social interaction and giving them the courage to believe in their own participation in the future. In this section we will present our findings of the three overreaching and interrelated themes that emerged from the interviews: 1) Building body capital for independence 2) Building body capital to maintain vitality and being in control 3) Building resources for social interaction.

Participants’ quotes are presented in italics and quotation marks refereeing to numbers (no) (see Table 2).

### Building body capital for independence

The importance of exercise to create a resource or capital to meet everyday challenges was clearly stated in many of the women’s accounts given during the interviews. They realized the importance of the group exercise program for disease management, and appeared to understand the factors that may aggravate symptoms and which are “*effective in managing symptoms and descries their risk of poor health”* (Woman no 4).

All of the women held the opinion that aging would lead to changes in health and body functions. Therefore, one of their concerns was to maintain the body’s physical function so as to be better prepared to meet these changes. One of the women who participated in the exercise explained it this way:

“*The motivation to participate is linked to health benefits, my feeling of independence and creating capability. As you age, you have an uncertain future ahead of you. I know I’m getting older. I do not want to think about all the obstacles that may occur in a few years’ time, which might make it difficult for me to attend [exercise program]*”. (*Woman no 2).* Another said *“The exercise helps me to have the energy to be social and engage in art and culture which I experience is of great importance to me at the moment as well as in the future. I really hope that the day I do not have the energy to participate is far ahead”.* (Woman no 12)

By exercising and being active, the women prevented physical deterioration. Through exercise, hey had all learnt that they became more confident about their own capacity and ability to achieve key objectives with regard to their quality of life. They said that it was important to strengthen arms and legs to prevent falls, and to facilitate a faster convalescence after illness.

The women’s investment in the body through exercises created a “*sense of energy*” (*Woman no 4 9)* and motivation all of the women to cope with everyday activities, as reflected in the following statements:

“*I need a body that gives me opportunities, so it is a good investment. Yes, in those periods when I have not been going to the gym, everything drops, both the initiative and ... Those periods when I have not attended due to illness have been difficult; 2006 just was such a horrible year. Exercise helps to keep me from being dependent on others or getting sick*” (Woman no 6). “*I have more assets, yes, we do not expect that, but taking a little care of our bodies and staying in shape pays off. Of course, it enables one to continue to go out and get access to different types of experiences. I can stay on my feet longer”*. (Woman no 1)

Being aware of the inevitable change in their physical abilities, all these women met those changes by upholding their lifelong habits of physical activity so as to ensure continued independence.

### Building body capital to maintain vitality and being in control

The exercise was described by the women as giving them “*strength to initiate things themselves*,” (Woman no 10) and that they no longer remained apathetic because of an untrained body. The advantage of having a “*vital body*,” (Woman no11) as one put it, was that it contributed to self-initiated opportunities for activities and ventures for all the women. Others said “*the body becomes more alive*” (Woman no 3) when they are doing exercise and that they all felt they had more power in the execution of their daily tasks and keep health promoting habits. According to the women, this surplus of energy gave other benefits too, such as a good humor and enthusiasm for social events. As one said:

*The body is important for me as a human being with regard to the physical and emotional experiences I get the energy to control my thoughts better. The exercise makes me more positive and therefore it gives additional energy to control emotions and not let me be overwhelmed by melancholy, which can happen. I think all people can get a little wistful at times, and it is then one needs energy to think about what opportunities one actually has in establishing good habits and to do something about it if one can. Yes, I feel that the training makes me more positive; I smile more and stay in a better mood. In addition, this means that I receive positive feedback from others*. (Woman no 1)

Through regular exercise, the women were able to maintain their bodily physical agility, which in turn meant that they acquired body capital and achieved several gains as described here: “*The training gives me a little energy and makes me move both physically and mentally*.” (Woman no 3) To these women, engaging in training appears to help them keeping their motivation for continued physical activities by generating emotional well-being and a sense of control or being empowered. Another said: “*The exercises allow me to experience a connection between body and mind. Helps to reduce distress and sadness. The exercises improve my self-esteem and attention as well as provides a sense of belonging. I think the exercises contribute to good health and maintain my… - I experience that I’m able to do things that are important to me (Woman no 8).*

### Building body capital for social interaction

The physical training of the body had a positive, emotional gain for all the women. Some of them recounted, for example, that training “*gives an appetite for life,”* (Woman no 8) and the additional energy surplus they experienced from the training allowed them to be socially more active. The group exercise constitutes an arena for contact with other people*.* One woman said: *Will simply make contact with people - I feel better about myself - Maybe I’m better embedded in my own body - I actually have the profits to contact and to get involved with other people* (Woman no 7).

By exercising, the women achieved other types of capital such as social capital in terms of social participation and being together with friends. It was their experience that a trained body meant they felt safer, they could be physically more active and consequently participate more in social situations with other people. The following account illustrates this experience:

*“I believe that the training enables me to move more outside the home and the outdoor life promotes social participation. I see myself as a fighter. That means it is about not giving up about rewarding oneself for the efforts. The social benefit is what you gain from being with others, and this is not obvious when one is old. I’m curious about other people, very curious”*. (Woman no 14)

The effort they put into exercise to have a well-trained body also contributed to the women feeling physically attractive in addition to creating an energy surplus and better opportunities for social participation. It appears that some invested in their body to maintain physical functions with the purpose of increasing their external attraction towards others. One participant has experienced receiving positive feedback about her body and describes it this way:

*I am a vigorous and graceful adult woman who is relatively flexible and strong. Moreover, I get a lot of praise for it, not least from those who do not know that I am training, but who guess that I’m engaged in something. It’s nice, I actually like it very much that I’m visible* [laughs]. (Woman no 13)

By feeling physically strong through the exercise, the women also gained a “*sense of safety*” (Woman no 15) and could “*easily engage in social activities”* (Woman no 16). In addition, the exercise contributed to the women’s physical attractiveness and well-being, which might contribute to socially activity.

## Discussion

This aim of this study is to provide a deeper understanding of how women more than 70 years of age experience their regular attendance at group exercise classes with a program described consisting of weight bearing resistance, balance and stretching exercises, incorporating dancing steps and movements. We have explored how these women engage in decision making, self-surveillance and risk reduction practices and how they create meaning through physical activity. The main theme emerging from the data is the idea of ‘body capital’ as an important resource in old age. Body capital in this context is a resource-oriented concept and includes different types of knowledge, skills and awareness of opportunities. The body seems to be an important center of the women’s own experience of their health and capabilities. Our findings reveal that the women participating in the training program have an enduring passion for physical activity. Doing physical exercise on a regular basis is something that appears to be incorporated in their bodies and has become a habitual lifestyle. The study by Del Castillo [20] found that participation in physical activity early in life promotes participation late in life and confirms the results of several previous studies. On reaching old age, in line with the precepts of continuity theory, those individuals who had participated earlier in life are more likely to continue to be active [32, 33]. This lifestyle is what we have termed the accumulated cultural capital which gives access to socially desirable health gains [21]. One of our key findings is therefore the accumulation of body capital that arises from this lifelong physical exercise.

One of the most salient findings is the participants’ attribution of importance to physical activity for inclusion in societal arenas. They appear to look at being in and maintaining their position in a social network as a crucial motivation for investing in body capital [23, 24] and successful aging [18]. This is in line with studies that give evidence to older people ‘s desire to avoid invisibility and possible exclusion from social network by building body capital and their physical capabilities [34]. In that sense, Bourdieu’s perspective of capital illustrates the societal aspects of the training group classes. Group training may therefore, through physical activity and accumulated body capital, facilitate cultural resources such as social inclusion and belonging [21]. Accordingly, having a strong social network is indicative of regular physical activity and well-being. Engaging in physical activity maybe important for social networking for older people, as it seems to go along with higher physical functional status and good health throughout old age [35–37].

In addition, the women in this study told us about maintaining their bodies’ ability and expressing their feelings of satisfaction with their bodies. Although they mentioned some events that may contribute to a sense of diminished body capital, e.g. loss of a breast due to cancer treatment, they were mainly concerned with intrinsic motivational factors for doing exercise which, in line with Antoninetti and Garrett [23], is linked to the adaptations to biological changes and to individual and societal attitudes toward aging.

Imbalance between capacity of body and the environment may diminish a person’s physical capabilities and thereby body capital. Consequently, diminished body capital may result in a feeling of diminished self-worth [23]. The state of diminished body capital resonates with the women in our study’s statements about avoiding decay, and that exercise might be a way of tackling this expected imbalance. This might be related to the stated fear of being dependent and a burden on family and relatives. Physical autonomy appears, therefore, to be a core motivation for the maintenance of their body, which is also in accordance with the findings in other studies [24, 38]. In contrast, by doing exercise the women increase their body capital and thereby build up resistance to negative cultural forces and stereotypes linked to cultural perceptions and attitudes towards aging [23, 38]. As a consequence, several of them talked about the social benefit and the positive feedback they got for being fit and doing regular exercise.

The women experience a sense of energy surplus when they engage in physical activities in the group exercise program. Not only do they gain strength to execute daily activities, but they also report emotional gains from their physical activity in old age and express a positive attunement to aging. Twigg [39] might be urging students of gerontology to pay more attention to what the women in our study have said. She stresses the need to take into account and explore the more enjoyable sensations of the body. Strategies for effective communication in the sense of a joyful and attractive presentation to promote physical activity is also addressed by Ory et al. [40]. The results from the participants in our study, who had invested body capital through regular physical activity, with the purpose of profiting both emotionally and physically, give testimony to the importance of reasserting agency in studies of older people and their bodies. The apparent inconsistency with on the one hand accepting social stereotypes, but on the other fighting back probably has to do with differences in age. Whereas participants in our studies are older than 70, other studies, which tend to focus disproportionally on ideals of appearance, may target some younger persons or a specific gender, as is the case in the studies of Clarke 398 and Griffin [41].

Moreover, Segar et al. [42] found in their study that women who were primarily engaged in physical activity to enhance body shape were significantly less physically active than those with non-body shape motives. Thus, individual preferences towards physical activity illustrate the importance of studying the motivations for doing exercises. In comparison, the women in our study seemed not to have the body shape motivation but were more motivated by health gains and well-being. Furthermore, the women in our study appear to be strongly motivated for bodywork and they have been investing in their bodies for almost the whole of their lives.

Such investment in body capital might improve the women’s body image and increase self confidence, and ultimately result in feelings of empowerment [43]. The women’s investment in their body is likely to be associated with their socio-economic status. The higher an individual’s socio-economic status, the greater their opportunities to be physical active in old age [44–47]. Although retirement have negative impact on level of physical activity, high level of education as well as cultural’, physical’ and ‘economic’ capital are associated with increased involvement in physical activity. These individuals are likely to invest a considerable amount of time and money in activities designed to maximize the potential production and conversion of physical capital [44–47]. The women’s experiences reveal their awareness of doing exercises to create *direct* health benefits such as prevent physical deterioration and having creating a sense of energy and *derived* health benefits such as being independent and increase attractiveness. Our findings thus contrast with previous studies highlighting primarily negative body images among older women describing the body as; ‘problematic’ , ‘ugly’ , ‘awful’ , ‘the body as a disaster’ [43]. Nevertheless, one particular study [48] supported the idea that women report overall contentment with their bodies and simultaneously express dissatisfaction with their bodies and desire changes. The women in our study focus on meaningful training exercises to improve energy and they invest in their bodies to get opportunities to be independent, strong, flexible, and visible and have more control over their emotions. In addition, our study reveals one of the women’s prime motive for exercising. They have been doing training for many years and all of them talked about it as an enjoyable activity.

They engage in physical activity regularly spending their time and effort, and thus appear to intrinsically motivated. Titze et al. [49] found that individuals who enjoyed exercise were more likely to continue, may explain these finding. Similarly, Ferrand et al. [50] found that a high level of autonomous motivation and self-imposed discipline with respect to exercise are positively linked to well-being. These findings confirm the assumption that there is a growing trend for older people, rather than being *“over the hill”,* to be *“taking the hill by storm”* [17].

### Strengths and limitations

This study has limitations regarding transferability. First, our sample may not reflect the views of the wider population living at home. Second, this study was conducted in an urban area and may not be generalizable to more rural settings or other populations. Furthermore, the sample consists of women who have been engaging in group exercise, not individual exercise, which most likely makes a difference for their motivation regarding participation [51].

A strength of this study is that we apply a qualitative approach, which contributes to greater depth of understanding by exploring the subjective experiences of older women participating in a training program. This study is also unique in applying a theoretical framework based on cultural capital and health, with a specific focus on women aged 70 years or over and their experiences of participating in an exercise class.

Furthermore, in qualitative studies, the role of the researchers as producers of knowledge is important. Thus, we realize that our own preconceptions of exercise and physical activity in older age might have influenced our interpretations since all of us had positive experiences with exercise. However, all the women were asked questions about negative experiences with exercise, but nobody reported negative experiences.

## Conclusions

This study has provided insights into the older women’s understanding and experiences of group exercise in order to cope with the challenges of everyday life within a theoretical framework of cultural capital and health. The women had created cultural health capital, and more specifically body capital, by participating in the group exercise classes. Hence, the women’s investment in body capital through regular exercise facilitated social participation and maintaining their bodily strength. The women’s ability to extract *direct* health gains, such as strong bodies and attractive bodies, and *derived* health gains, such as being independent and enjoying social inclusion, gave testimony to the importance of physical activity in old age. The concept of cultural capital as an analytic tool seemed to uncover some of the motivational forces for the women participating in group exercise classes, and to show how their cultural resources, skills, dispositions and interactional styles influence their ability to obtain important abilities to maintaining and create active lifestyles.

Our findings might be of importance in shaping health promotion interventions for older people. There is a need for a consistent public information message to clearly communicate to the public about the benefits of exercise both on general health, well-being and social participation for older people.
